# Prevalence and Correlates of Fearing a Partner During the COVID-19 Pandemic in Britain: Findings from Natsal-COVID

**DOI:** 10.1007/s10896-023-00665-w

**Published:** 2023-11-24

**Authors:** Malachi Willis, Clare Tanton, Anne Conolly, Andrew J. Baxter, Raquel Bosó Pérez, Julie Riddell, Emily Dema, Andrew J. Copas, Wendy Macdowall, Chris Bonell, Catherine H. Mercer, Pam Sonnenberg, Nigel Field, Kirstin R. Mitchell

**Affiliations:** 1 MRC/CSO Social and Public Health Sciences Unit, University of Glasgow, Clarice Pears Building, 90 Byres Road, Glasgow, G12 8TB UK; 2 Faculty of Public Health and Policy, London School of Hygiene and Tropical Medicine, London, WC1H 9SH UK; 3 Institute for Global Health, University College London, London, WC1E 6JB UK; 4 NatCen Social Research, London, EC1V 0AX UK

**Keywords:** Intimate partner violence, Fearing partner, Domestic abuse, COVID-19, Pandemic, Cross-sectional survey

## Abstract

**Purpose:**

The COVID-19 pandemic and lockdown restrictions introduced personal and relationship stressors that potentially increased the risk of intimate partner violence (IPV) for some. We estimated the population prevalence and correlates of fearing a partner in the first year of the pandemic in Britain.

**Method:**

We used data from Natsal-COVID Wave 2—a web-panel survey undertaken one year after the initial British lockdown from 23 March 2020. Quotas and weighting were used to achieve a quasi-representative sample of the general population. Participants were asked about fearing a partner, which is a simple and valid screening tool to identify IPV experiences.

**Results:**

In our sample (unweighted *n* = 6302, aged 18–59), 9.0% of women and 8.7% of men reported fearing a partner in the first year of the pandemic. Women (73.3%) were more likely than men (49.9%) to indicate that fearing a partner made them feel anxious or depressed; men were more likely to report increased substance use (30.8% vs. 18.4%) and affected work/studies (30.0% vs. 20.0%). For both women and men, fearing a partner during the first year of the pandemic was associated with established health and wellbeing outcomes like anxiety/depression, alcohol use, accessing sexual/reproductive health services, and relationship dissolution as well as feeling that the “pandemic made things worse” across various life domains.

**Conclusions:**

Population-level estimates of IPV during the COVID-19 pandemic highlight harmful experiences that occurred alongside other wide-ranging hardships, and the associations presented identify key populations with potential ongoing need. We make recommendations for primary, secondary, and tertiary prevention of IPV.

## Introduction

Defined as sexual violence, physical violence, stalking, or psychological aggression (including coercive tactics) by a current or former partner, intimate partner violence (IPV) occurs commonly across the world (Breiding et al., 2015; Willis & Marcantonio, 2021). In the UK context, for example, a cross-sectional study of adults aged 18–64 in London found that 64.9% of women and 62.3% of men reported experiencing psychological aggression from a partner in the past year; other types of IPV victimization included sexual coercion (23.3% of women, 18.0% of men) and physical assault (17.0% of women, 15.9% of men) (Costa et al., 2015). Experiencing IPV can be detrimental to a person’s mental, physical, sexual, and reproductive health (Dillon et al., 2013; Laskey et al., 2019).

In 2020, concerns that levels of IPV would be exacerbated by the COVID-19 pandemic quickly spread (Landoni & Chiara, 2020). Public health measures intended to mitigate SARS‑CoV‑2 transmission (e.g., physical distancing, at-home isolation) alongside heightened stress levels may have incidentally created environments in which intimate relationships were under duress and IPV risk increased (Bosó Pérez et al., 2021; Peterman et al., 2020). In the UK, preliminary evidence from an Office for National Statistics (ONS) report found there were 65% more calls to the National Domestic Abuse Helpline during the first three months of the initial national lockdown (announced on 23 March 2020) compared with the first three months of 2020 (Stripe, 2020). Relatively smaller estimates regarding increased usage of domestic violence hotlines have been published in other countries that adjusted for seasonal variation in number of calls (e.g., comparing January–May 2021 with January–May 2020); there was a 32% increase in calls in Argentina (Perez-Vincent et al., 2020) and a 7.5% increase in the US (Leslie & Wilson, 2020). Increased use of such hotlines does not necessarily imply greater prevalence of IPV; these data may instead reflect heightened severity of IPV experiences or fewer coping, help-seeking, and fleeing options available due to isolation measures.

Population estimates of self-reported prevalence of IPV, as well as its antecedents and consequences, may help better understand people’s experiences within relationships during the COVID-19 pandemic. An online general population quota-based cross-sectional survey of cisgender women and trans people in a northern US state found that levels of reporting any type of IPV in the three months before the COVID-19 pandemic (16.2%) were similar to the three to five months following the onset of lockdown restrictions (15.1%); 9.7% of those who reported IPV since the start of pandemic-related measures indicated that they had experienced new, worse, or increased violence compared with pre-pandemic IPV experiences (Peitzmeier et al., 2021). As part of the International Sexual HeAlth and REproductive Health (I-SHARE) study, participants across 30 countries (not including the UK) completed an online cross-sectional survey that included an IPV assessment. Preliminary results suggested that 9.2% of participants reported experiencing physical or sexual IPV in the three months preceding lockdown measures related to COVID-19 compared with 7.0% in the three months after such measures were introduced (Campbell et al., 2021). Otherwise, self-reported data on IPV and its consequences during the first year of the COVID-19 pandemic are lacking in large population studies—no such studies to our knowledge have been conducted and published on the British general population.

Using data from the Natsal-COVID Wave 2 survey, the present study had three research aims. First, we estimated the prevalence of fearing a partner in Britain during the first year of the COVID-19 pandemic, using this as a screening tool for exposure to IPV. Previous research has shown that a single question about fear of a partner is a simple and valid way to identify IPV victims (Signorelli et al., 2022). This single-item approach demonstrated moderate sensitivity (i.e., 65% of women identified by the Composite Abuse Scale [CAS] as having been exposed to IPV in the past 12 months reported “yes” to fearing a partner in the past 12 months, high specificity (i.e., 95% of women not identified as IPV victims by the CAS reported “no”), and a robust area under the receiver operation curve (i.e., 0.8). Second, we evaluated the self-reported consequences of fearing a partner during the pandemic. Finally, we examined whether established health and wellbeing correlates of IPV were associated with reported fear of a partner during the pandemic. For both self-reported consequences and correlates, we predicted that those who reported fearing a partner would have negative experiences across various areas of their life (i.e., physical health, mental health, substance use, relationships, daily life).

## Method

The Natsal-COVID Wave 2 survey was conducted from 27 March to 26 April 2021 (i.e., approximately 12 months after the first lockdown measures in Britain were announced in March 2020). This web-panel survey was administered by Ipsos and assessed many aspects of sexual, reproductive, and relational health (average survey length: 13 min). Approximately 150,000 panellists, including those from Wave 1 who were willing to be re-contacted, were contacted via email, and 38,731 started the survey—of these, 11,708 were ineligible or did not provide consent, 17,230 were diverted from completing the survey because their quota was full, 2,376 abandoned the survey before completion, 490 failed quality checks, and 269 experienced a technical error. In sum, 6,658 participants completed the survey; the target sample size for Wave 2 was 6,000 people aged 18–59 with an additional boost of 500 people aged 18–29.

The analytic sample included all participants who responded to the fear of a partner item and comprised 6302 participants (3370 women, 2932 men^1^). To achieve a quasi-representative sample of the British population, we used quotas of age, gender, region, and social grade and subsequently weighted the data to match distributions for the quota characteristics, ethnicity, and sexual identity that would be expected based on probability sample surveys (i.e., the Annual Population Survey, Health Survey for England, and Natsal-3; Dema et al., 2022). For example, the weighted distribution of ethnicities in our sample (85.7% White, 8.1% Asian/Asian British, 3.4% Black/Black British, 1.7% mixed/multiple, and 1.1% other) closely aligned with general population estimates for those aged 18–59 in the UK (84.3%, 8.5%, 3.7%, 1.3%, and 2.1%, respectively). We obtained ethical approval from the University of Glasgow Medical, Veterinary and Life Sciences College Ethics Committee (reference 20,019,174) and London School of Hygiene and Tropical Medicine Research Ethics committee (reference 22,565). See Dema et al. (2022) for further methodological details.

### Measures

#### Fear of a Partner

Introducing the set of questions related to IPV^2^ (which appeared toward the end of the survey), the questionnaire instructed participants to think about experiences they “may have had with violent or controlling behaviours.” Acknowledging that IPV may take many forms—including verbal or physical, we asked about lifetime experiences of fearing a partner using the following question: “Has a partner or ex-partner ever made you feel afraid of them based on their words or their actions?” Participants who reported ever fearing a partner were asked to indicate the frequency in the past 12 months: “Not at all,” “Once,” “A few times,” or “Many times.” Those who reported fear of a partner at least once in the past 12 months (i.e., in the first year of the pandemic) were asked to indicate whether that experience had affected them in the any of the following ways (selecting all that applied): “It damaged my physical health,” “It made me feel anxious or depressed,” “It made me drink more alcohol/take more drugs,” “It affected my work or studies,” “It affected my relationship with my children,” “It affected my relationships with friends or other family members,” “It affected the way I go about my day-to-day life,” “It had another effect,” “It didn’t really affect me.” In addition to evaluating these consequences individually, we created a summed score to determine whether participants reported experiencing more than one consequence of fearing a partner in the past year.

#### Sociodemographic Variables

Participants were categorized according to sociodemographic characteristics of interest. Age in years comprised five groups: 18–24, 25–29, 30–34, 35–44, and 45–59. Women who have sex with women (WSW) and men who have sex with men (MSM) were defined as those reporting at least one same-sex sexual partner in the past five years—consistent with previous Natsal publications (e.g., Mercer et al., 2013). We defined three distinct relationship statuses: cohabiting relationship (“in a relationship and living together”), non-cohabiting relationship (“in a relationship and not living together”), and “single.” Having children in the home during the pandemic was defined as “living with child family members aged under 18.” Social grade was grouped into three categories: higher (“A – Upper middle class” or “B – Middle class”), median (“C1 – Lower middle class” or “C2 – Skilled working class”), and lower (“D – Working class” or “E – Lower level of subsistence”).

#### Health and Wellbeing Variables

Measures related to current health and wellbeing included general health, mental health, and substance use. Participants rated their general health and were grouped dichotomously: poor (“Very bad” or “Bad”) versus the other response options (“Fair,” “Good,” or “Very good”). Participants reported frequency of their anxiety symptoms in the past two weeks via the Generalized Anxiety Disorder 2-item (i.e., “Feeling nervous anxious or on edge” and “Not being able to stop or control worrying”) and their depression symptoms via the Patient Health Questionnaire-2 (i.e., “Little interest or pleasure in doing things” and “Feeling down, depressed or hopeless”), respectively. Response options ranged from (0 “Not at all” to 3 “Nearly every day”). Scores were summed for each pair of items; participants were identified as having experienced anxiety or depression symptoms over the past two weeks if they scored at least a 3 on the GAD-2 (Kroenke et al., 2007) or PHQ-2 (Kroenke et al., 2003). They also reported on how many days in the past week they had consumed an alcoholic drink as well as whether their alcohol consumption had changed compared with before the COVID-19 pandemic (“More these days,” “About the same,” or “Less these days”); we focused on those participants who reported consuming alcohol 4 + days in the past week (a frequency associated with increased mortality; Hartz et al., 2018) or having increased their alcohol usage in the past year.

The questionnaire also asked participants whether they had accessed sexual or reproductive health (SRH) services for themselves. They were instructed to include phone, online, or video appointments. Response options from which participants could select all that applied were “Contraception services/advice,” “Fertility services/advice,” “Maternity/antenatal services” (women only), “Abortion/Pregnancy termination services” (women only), “Cervical screening (smear test/pap test)” (women only), “STI (Sexually Transmitted Infection) testing,” “STI follow-up care,” “HIV testing,” “Advice or counselling for sexual problems,” “Relationship support services/advice,” “Sexual assault/rape support services or helplines,” “Other type of sexual or reproductive health service/advice,” and “None.” For our primary analyses, we assessed whether participants accessed any service; see Appendix Table 3 for separate analyses on individual SRH services.

Finally, participants reported whether they had “experienced the breakdown of a romantic or sexual relationship since the start of the first lockdown” (“Yes” or “No”) as well as whether they had moved homes or the people in their home changed during this period (versus “There have been no changes to who I live with”).

#### Pandemic-Related Effects

Participants who reported fearing a partner in the past 12 months were asked, “Did the coronavirus pandemic, or related restrictions, play a role in the experience(s) you have told us about?” Response options included were “The pandemic made things worse,” “The pandemic made no difference,” or “The pandemic made things better.” The questionnaire also asked participants if they perceived the pandemic to have had a negative effect on various aspects of their life. They could select all that applied from a list of effects, which we categorized into three domains: their intrapersonal life (i.e., “My physical health,” “My mental health,” “My well-being”), their interpersonal life (i.e., “My romantic or sexual relationships,” “My relationships with friends or family,” “My relationships with neighbours), or their daily life (i.e., “My work,” “My education,” “My household finances,” “My caring responsibilities,” or “My access to groceries, medication, or essentials”).

### Analysis

**Fig. 1:**
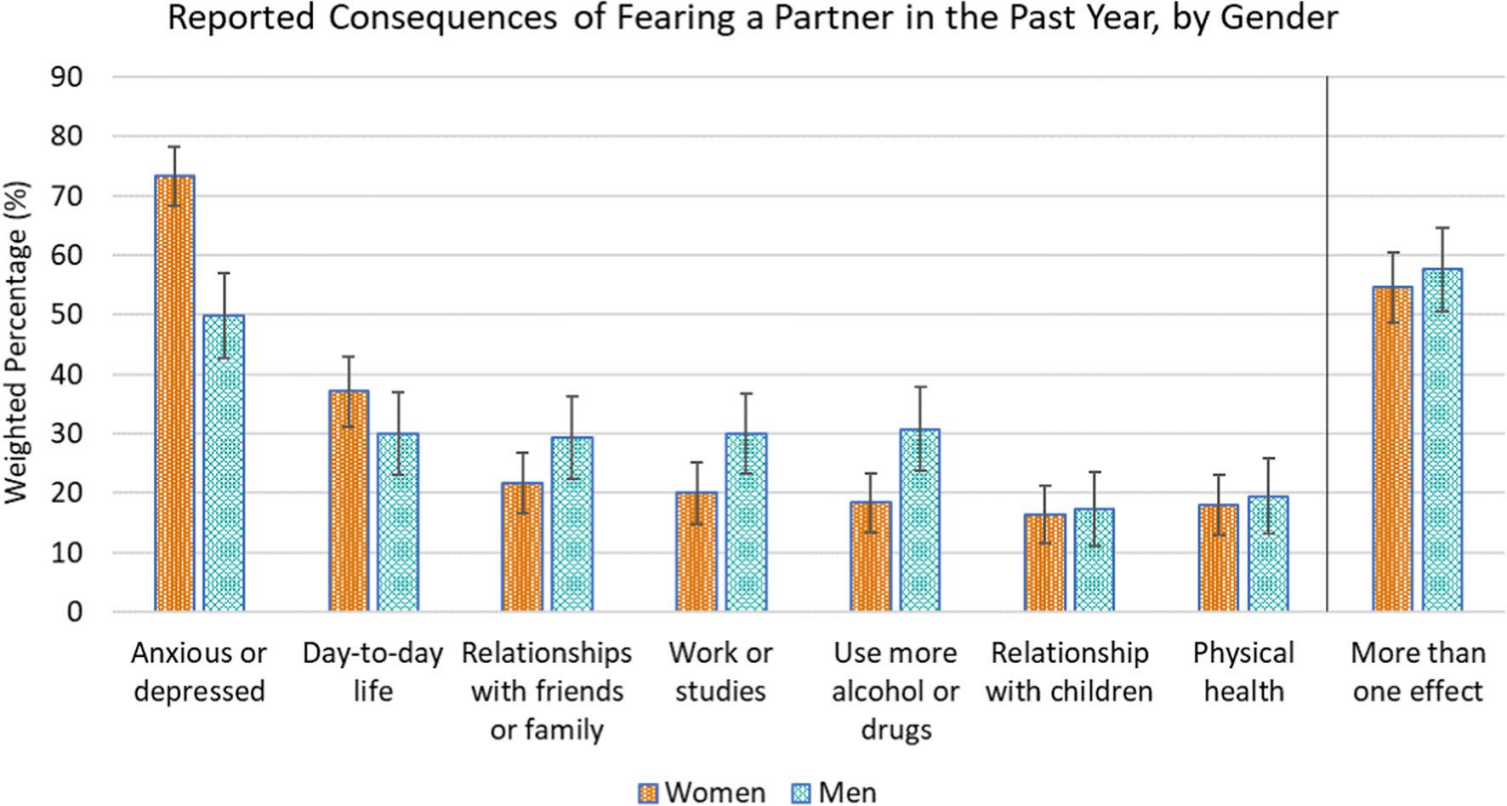
Weighted percentages of women (unweighted *n* = 320) and men (unweighted *n* = 247) reporting consequences of fearing a partner in the past year

We used Stata’s (version 16.1) complex survey analysis functions to incorporate weighting and stratification. In response to the item measuring our primary variable of interest (i.e., reported fear of a partner in the past year), 2.0% of women (unweighted *n* = 65) and 1.4% of men (unweighted *n* = 43) indicated “prefer not to say;” we removed these participants and did not account for their missing data in our analyses. Due to the gendered nature of interpersonal violence, descriptive statistics for prevalence and frequency of fearing a partner in the first year of the pandemic are presented by gender and further broken down by other key sociodemographic characteristics (Table 1). Self-reported consequences associated with fearing a partner are presented by gender (Fig. 1). We compared prevalence of correlates of IPV between participants who reported fearing a partner and those who did not (Table 2). We complemented each set of descriptive statistics with odds ratios obtained by conducting logistic regression models, first adjusting for age (as a continuous variable) and then for age and relationship status. We provided 95% confidence intervals for odds ratios and indicated whether they were statistically significant (α < 0.05).

**Table 1:** Weighted percentages of women and men reporting fear of a partner in year following the first lockdown in Britain in March 2020

	Feared partner in past year	Once	Few times	Many times	Denominator (unw, w)	aOR^a^	*p*
Women *95% CI*	9.0 (8.1-10.1)	2.3 (1.8-2.9)	5.0 (4.3-5.8)	1.7 (1.3-2.3)	3370, 3161		
Age							< 0.001
18-24 years *95% CI*	16.1 (12.8-20.1)	2.9 (1.6-5.0)	10.1 (7.4-13.5)	3.2 (1.9-5.3)	493, 390	5.58 (3.55-8.77)	
25-29 years *95% CI*	11.4 (8.8-14.7)	2.3 (1.3-4.2)	7.1 (5.1-9.8)	2.0 (1.0-3.9)	557, 453	5.74 (3.67-8.99)	
30-34 years *95% CI*	12.7 (9.7-16.5)	2.7 (1.4-4.9)	7.4 (5.1-10.6)	2.7 (1.5-4.8)	442, 368	2.78 (1.52-5.07)	
35-44 years *95% CI*	8.2 (6.5-10.4)	2.4 (1.5-3.7)	4.2 (3.0-5.9)	1.6 (0.9-2.8)	798, 746	2.47 (1.58-3.87)	
45-59 years *95% CI*	5.2 (4.0-6.8)	1.9 (1.2-3.1)	2.3 (1.5-3.4)	1.0 (0.5-1.8)	1080, 1205	ref.	
Same-sex partner							< 0.001
In past 5 years *95% CI*	28.8 (20.4-38.9)	9.7 (4.9-18.1)	11.0 (6.1-19.0)	8.1 (3.6-16.9)	163, 81	4.41 (2.87-6.75)	
Not in past 5 years *95% CI*	8.4 (7.5-9.5)	2.1 (1.6-2.7)	4.8 (4.0-5.6)	1.6 (1.2-2.1)	3130, 3005	ref.	
Relationship status							< 0.001
Cohabiting rel. *95% CI*	8.8 (7.6-10.2)	2.6 (1.9-3.5)	4.7 (3.8-5.8)	1.5 (1.0-2.2)	2004, 1939	3.00 (1.94-4.65)	
Non-cohabiting rel. *95% CI*	11.5 (8.8-14.9)	2.2 (1.2-4.0)	7.9 (5.6-10.9)	1.4 (0.7-3.0)	464, 407	3.63 (2.17-6.10)	
Single *95% CI*	6.5 (5.0-8.5)	1.6 (0.9-2.8)	3.3 (2.2-4.9)	1.6 (0.9-2.7)	822, 742	ref.	
Children at home							< 0.001
Yes *95% CI*	12.4 (10.4-14.7)	3.6 (2.5-5.0)	6.8 (5.3-8.6)	2.0 (1.3-3.1)	1026, 979	1.70 (1.32-2.20)	
No *95% CI*	7.5 (6.5-8.7)	1.7 (1.3-2.4)	4.1 (3.4-5.1)	1.6 (1.2-2.3)	2341, 2179	ref.	
Social grade							0.002
Lower - D/E *95% CI*	12.7 (10.6-15.2)	2.0 (1.2-3.3)	8.5 (6.7-10.6)	2.2 (1.4-3.4)	866, 782	1.81 (1.28-2.58)	
Middle - C1/C2 * 95% CI*	8.3 (7.0-9.8)	2.6 (1.9-3.6)	4.4 (3.5-5.6)	1.3 (0.8-2.0)	1677, 1659	1.27 (.90-1.78)	
Upper - A/B *95% CI*	6.7 (5.2-8.7)	1.8 (1.1-3.0)	2.6 (1.7-3.9)	2.3 (1.4-3.7)	827, 721	ref.	
Men *95% CI*	8.7 (7.6-9.9)	2.0 (1.5-2.7)	4.6 (3.8-5.6)	2.0 (1.5-2.7)	2932, 3129		
Age							< 0.001
18-24 years *95% CI*	16.4 (12.6-21.0)	4.5 (2.6-7.6)	8.8 (6.0-12.7)	3.1 (1.7-5.7)	430, 482	3.49 (2.37-5.13)	
25-29 years *95% CI*	16.8 (13.0-21.4)	4.5 (2.7-7.3)	7.4 (5.0-10.9)	4.8 (2.8-8.1)	370, 381	2.35 (1.57-3.50)	
30-34 years *95% CI*	8.9 (5.6-13.9)	2.0 (0.8-4.9)	3.2 (1.5-7.0)	3.6 (1.7-7.7)	239, 282	2.65 (1.75-4.00)	
35-44 years *95% CI*	8.0 (6.0-10.5)	1.6 (0.9-2.9)	5.0 (3.5-7.2)	1.3 (0.7-2.5)	703, 807	1.62 (1.11-2.37)	
45-59 years *95% CI*	3.4 (2.5-4.7)	0.5 (0.2-1.1)	2.1 (1.4-3.2)	0.8 (0.4-1.6)	1190, 1175	ref.	
Same-sex partner							< 0.001
In past 5 years *95% CI*	30.1 (22.4-39.1)	9.2 (5.3-15.5)	13.4 (7.6-22.5)	7.5 (4.1-13.3)	254, 132	3.85 (2.38-6.22)	
Not in past 5 years *95% CI*	7.6 (6.5-8.8)	1.7 (1.2-2.4)	4.2 (3.4-5.1)	1.7 (1.2-2.4)	2628, 2945	ref.	
Relationship status							0.009
Cohabiting rel. *95% CI*	9.0 (7.5-10.6)	2.2 (1.5-3.1)	4.8 (3.8-6.1)	2.0 (1.3-2.9)	1709, 1815	1.66 (1.20-2.30)	
Non-cohabiting rel. *95% CI*	16.2 (12.3-21.1)	3.5 (2.0-6.1)	8.0 (5.3-11.9)	4.7 (2.7-8.1)	343, 382	1.59 1.05-2.43)	
Single *95% CI*	4.2 (2.9-6.0)	1.2 (0.6-2.3)	2.2 (1.3-3.7)	0.8 (0.3-2.0)	804, 845	ref.	
Children at home							0.461
Yes *95% CI*	8.5 (6.6-10.9)	1.2 (0.6-2.2)	5.5 (4.0-7.5)	1.8 (1.0-3.2)	780, 880	1.13 (0.82-1.57)	
No *95% CI*	8.7 (7.4-10.2)	2.4 (1.7-3.2)	4.3 (3.4-5.5)	2.0 (1.4-2.8)	2141, 2234	ref.	
Social grade							0.560
Lower - D/E *95% CI*	9.4 (7.2-12.3)	.5 (2.1-5.6)	4.7 (3.2-6.9)	1.3 (0.6-2.5)	690, 732	0.92 (.63-1.33)	
Median - C1/C2 *95% CI*	8.0 (6.5-9.9)	1.0 (0.6-1.8)	4.6 (3.5-6.2)	2.4 (1.6-3.6)	1203, 1659	0.84 (.61-1.16)	
Higher - A/B *95% CI*	9.4 (7.7-11.4)	2.9 (2.0-4.1)	4.6 (3.4-6.1)	2.0 (1.3-3.1)	1039, 737	ref.	

^a^Age-adjusted odds ratio

**Table 2:** Weighted percentages of health and wellbeing variables and associations with fearing a partner in the past year, by gender

	No partner fear in past year	Feared partner in past year	aOR^a^	*p*	aOR with rel. status^b^
Women					
General health (Bad/Very bad)	6.6 (5.7–7.6)	10.5 (7.4–14.7)	1.95 (1.28–2.95)	0.002	1.95 (1.24–3.06)
Anxiety symptoms in past two weeks	31.1 (29.4–32.9)	57.5 (51.5–63.3)	2.54 (1.96–3.30)	< 0.001	2.62 (1.99–3.44)
Depression symptoms in past two weeks	29.3 (27.-31.0)	53.3 (47.4–59.1)	2.54 (1.96–3.30)	< 0.001	2.65 (2.02–3.47)
Drank alcohol 4 + days in past week	10.4 (9.3–11.6)	15.5 (11.7–20.3)	1.83 (1.28–2.61)	0.001	1.76 (1.22–2.54)
More alcohol use in past year	13.0 (11.8–14.3)	27.5 (22.6–33.0)	2.37 (1.77–3.16)	< 0.001	2.28 (1.69–3.08)
Accessed SRH services in past year	28.0 (26.3–29.7)	51.0 (45.0–57.0)	2.11 (1.62–2.74)	< 0.001	2.09 (1.60–2.74)
Relationship dissolution in past year	6.6 (5.7–7.6)	23.7 (19.1–29.1)	5.27 (3.77–7.36)	< 0.001	5.99 (4.05–8.87)
Moved or household changed in past year	19.8 (18.4–21.3)	41.2 (35.5–47.2)	2.29 (1.73–3.03)	< 0.001	2.27 (1.70–3.05)
Negative intrapersonal effect of pandemic	61.8 (60.0-63.7)	73.7 (68.1–78.7)	1.54 (1.16–2.06)	0.003	1.66 (1.23–2.25)
Negative interpersonal effect of pandemic	43.1 (41.2–44.9)	55.8 (49.8–61.7)	1.63 (1.26–2.10)	< 0.001	1.61 (1.23–2.09)
Negative daily life effect of pandemic	52.1 (50.2–54.0)	65.6 (59.7–71.0)	1.59 (1.22–2.08)	0.001	1.68 (1.28–2.22)
Denominator (unweighted, weighted)	3050, 2875	320, 286	—	—	—
Men					
General health (Bad/Very bad)	5.5 (4.6–6.5)	6.2 (3.6–10.3)	1.81 (1.00-3.29)	0.051	1.73 (0.93–3.25)
Anxiety symptoms in past two weeks	25.0 (23.1–26.9)	59.4 (52.3–66.2)	3.51 (2.55–4.83)	< 0.001	3.75 (2.68–5.24)
Depression symptoms in past two weeks	28.3 (26.4–30.2)	59.6 (52.6–66.2)	3.19 (2.33–4.36)	< 0.001	3.49 (2.51–4.84)
Drank alcohol 4 + days in past week	17.2 (15.7–18.8)	24.2 (18.9–30.3)	1.89 (1.34–2.65)	< 0.001	1.88 (1.33–2.67)
More alcohol use in past year	15.6 (14.2–17.2)	36.1 (29.9–42.9)	2.77 (2.02–3.80)	< 0.001	2.63 (1.90–3.64)
Accessed SRH services in past year	8.9 (7.8–10.2)	43.6 (36.7–50.7)	5.64 (3.97-8.00)	< 0.001	4.89 (3.39–7.05)
Relationship dissolution in past year	7.5 (6.4–8.8)	12.1 (8.4–17.0)	2.91 (1.82–4.65)	< 0.001	3.71 (2.23–6.18)
Moved or household changed in past year	19.2 (17.6–21.0)	57.3 (50.2–64.1)	4.11 (3.02–5.60)	< 0.001	3.98 (2.89–5.47)
Negative intrapersonal effect of pandemic	51.2 (49.1–53.3)	66.3 (59.3–72.6)	1.69 (1.23–2.32)	0.001	1.82 (1.32–2.53)
Negative interpersonal effect of pandemic	35.5 (33.5–37.5)	43.0 (36.3–50.1)	1.41 (1.04–1.90)	0.026	1.58 (1.16–2.16)
Negative daily life effect of pandemic	45.6 (43.5–47.7)	66.0 (59.0-72.3)	2.16 (1.58–2.96)	< 0.001	2.04 (1.8–2.81)
Denominator (unweighted, weighted)	2685, 2856	247, 272	—	—	—

^a^Age-adjusted odds ratio. ^b^Age-adjusted odds ratio controlling for relationship status

95% confidence intervals are provided in parentheses

## Results

### Prevalence of Fearing a Partner

Among 6654 participants, 9.0% of women (unweighted *n* = 320) and 8.7% of men (unweighted *n* = 247) reported fearing a partner during the first 12 months of the pandemic. Regarding frequency, 2.3% of women feared a partner once during this period, 5.0% a few times, and 1.7% many times (for men: 2.0%, 4.6%, and 2.0%, respectively). Lifetime prevalence estimates of fearing a partner were 32.9% for women and 14.7% for men.

Sociodemographic characteristics significantly associated with fearing a partner in the first year of the pandemic for women and men included being younger, having had a same-sex partner in the past five years, and being in a relationship (Table 1). For women but not men, living with children at home and being in a lower social grade were also significantly associated with fearing a partner.

### Self-Reported Consequences of Fearing a Partner

Overall, 91.0% of women and 93.7% of men who reported fearing a partner in the first year of the pandemic identified at least one negative consequence (Fig. 1). Over half of women (54.6%) and men (57.6%) reported being affected in multiple ways. Controlling for age and relationship status, women (73.3%) were significantly more likely than men (49.9%) to indicate that fearing a partner made them “feel anxious or depressed”, *p* < .001. Conversely, men were significantly more likely than women to report that this experience made them “drink more alcohol/take more drugs” (30.8% vs. 18.4%), *p* = .002, and “affected [their] work or studies” (30.0% vs. 20.0%), *p* = .014.

### Health and Wellbeing Correlates of Fearing a Partner

For health and wellbeing variables assessed, women and men who feared a partner in the first year of the pandemic reported experiencing worse outcomes during this time than those who did not fear a partner (Table 2). Controlling for age and relationship status, participants fearing a partner in the past year had greater odds of reporting anxiety and depression symptoms in the past two weeks and greater alcohol consumption (both in number of days in the past week and in general relative to before the pandemic). Those who feared a partner were also more likely to have accessed SRH services, experienced a breakup, and moved or changed households.

Although each of these associations were significant for both women and men, there were also some gendered patterns. Regarding the main effects of gender, women in general (i.e., including those who did or did not fear a partner in the past year) reported significantly greater anxiety symptoms than men in general (*p* < .001), less frequent alcohol use (*p* < .001), and greater likelihood of accessing SRH services (*p* < .001). There were two significant interactions between gender and fearing a partner. First, fearing a partner had a stronger effect on the likelihood of accessing SRH services for men than it did for women, *p* < .001. Second, men who feared a partner in the past year were particularly likely to have moved or changed households compared with women who feared a partner, women who did not fear a partner, and men who did not fear a partner, *p* = .009.

### Pandemic-Related Effects

When asked about the pandemic’s effect on their experiences of fearing a partner in the past year, significantly more women (49.8%) than men (41.4%) indicated that the pandemic worsened their experiences of fearing a partner, controlling for age and relationship status, OR = 1.54, 95% CI [1.05–2.27], *p* = .027. The negative influence of the pandemic extended to other areas of life for those who feared a partner in the past year; specifically, these participants were significantly more likely than those who did not fear a partner to have perceived the pandemic to have had a negative effect on their intrapersonal life, their interpersonal life, or their daily life (Table 2). Regarding main effects of gender, women were more likely than men to report negative pandemic-related effects on their intrapersonal life (*p* < .001), interpersonal life (*p* < .001), and daily life (*p* < .001); gender did not significantly moderate the associations of fearing a partner and these outcome variables. Descriptively, 38.7% of women and 25.4% of men who feared a partner in the past year reported that the pandemic negatively affected their life in all three domains measured—compared with 23.0% of women and 16.5% of men who did not fear a partner, respectively.

## Discussion

Using a quasi-representative sample of the British population, we found that one in 11 women and men reported fearing their partner in the year following the first COVID-19 national lockdown. However, this is likely an underestimate of IPV experiences during the pandemic because single-item measures of fearing a partner can miss up to 35% of IPV victims who may have otherwise been identified by gold-standard measures like the CAS (Signorelli et al., 2022). Our analyses suggested that groups at greater risk of reporting fear of a partner during this time included younger people, sexual minorities, and women of lower socioeconomic status. Corroborating these findings, preliminary I-SHARE data on IPV across 30 other countries during the COVID-19 pandemic indicated that (1) increasing age was associated with decreased odds of physical violence from a partner, (2) identifying as gay, bisexual, asexual, and pansexual were all associated with greater odds of sexual violence from a partner, and (3) having a worse economic situation during the pandemic was associated with greater odds of physical violence from a partner while having a better economic situation was associated with lower odds of sexual coercion from a partner (Campbell et al., 2021). And like the I-SHARE study, we did not find that the reporting of fearing a partner varied by whether people lived with their partner at the time of the study. Indeed, there is evidence that more than half of people in steady non-cohabiting relationships still engaged in intimate partner physical contact with people outside of their household during the first four months after the lockdown in Britain was announced (Sonnenberg et al., 2022). That said, fearing a partner may still have occurred without having been in the same physical space.

Although we did not find that the proportions of participants reporting fear of a partner in the first year of the COVID-19 pandemic varied by gender (9% of women and 9% of men), 59% of men who had ever feared a partner reported fearing a partner in the past year compared with 27.4% of women—reflecting women’s higher lifetime prevalence of fearing a partner. Helping contextualize this finding, men in the I-SHARE study were 1.8 times as likely as women to have experienced IPV during the pandemic (Campbell et al., 2021). Similar proportions of women and men in our study also reported negative consequences of fearing their partner, with women being more likely to experience anxiety and depression while men were more likely to have their professional lives affected and increase their substance use as a result. Further, our data suggested that women in lower socioeconomic positions or with children at home were particularly vulnerable; not only were they more likely to report fearing a partner, but women in these situations were likely not as able as men to relocate and leave a household in which they feared a partner—as evidenced by our finding that fewer women reported moving or changing household during the year in which they feared a partner. A final gender difference worth noting was that women were more likely to report that the pandemic itself worsened their experiences of fearing a partner as well as several other aspects of their life (i.e., intrapersonal, interpersonal, daily life), which contributes data to a collection of other studies demonstrating that women were more likely than men to experience negative outcomes directly linked to the COVID-19 pandemic (Oreffice & Quintana-Domeque, 2021;Porto & Quintana-Domeque, 2021).

Despite the significant difference in the proportion of women and men indicating that fearing a partner made them feel anxious or depressed, this negative mental health outcome was the most common self-reported consequence in our sample for both women and men. Further, men in our sample were about 3.5 times as likely (and women about 2.6 times as likely) to report anxiety and depression symptoms in the past two weeks if they had reported fearing a partner in the past year. Other mental health statuses consistently associated with experiencing IPV include posttraumatic stress disorder, suicidal ideation, eating disorder, and loneliness (Laskey et al., 2019). These robust associations demonstrate the strong ties between IPV and mental health, which illustrate the importance of prioritizing sexual and relational health in conceptualizations of broader well-being (Mitchell et al., 2021).

In addition to people’s self-identified consequences of fearing a partner, our findings indicated that fearing a partner in the first year of the COVID-19 pandemic was associated with more frequent alcohol use. Indeed, consuming hazardous levels of alcohol during the pandemic was associated with experiencing recent physical IPV in a sample of Ugandan women (Miller et al., 2022). Previous evidence has found that people may use alcohol to deal with problems associated with experiencing IPV (e.g., mental health concerns, sexual assault), which can lead to greater and lasting alcohol use without effectively helping them cope (Øverup et al., 2015). Interventions designed for IPV victims should help develop effective adaptive coping strategies to mitigate long-term consequences, which do not themselves cause harm.

Our quantitative findings on fearing a partner in Britain during the COVID-19 pandemic can be further contextualized by considering qualitative findings from the Natsal-COVID study. In a qualitative analysis of 19 people’s experiences of relationship difficulties, Bosó Pérez et al. (2021) found that pandemic-related stress (manifesting in problems like financial strain, health concerns, and sex life issues) negatively affected how well couples could adapt—which led to violence for some and intensified violence for others if already present. Indeed, a national sample in the US found that men as well as women were more likely to perpetrate IPV if they had experienced a stressful life event (e.g., economic stress due to a financial crisis or changes in job responsibilities, interpersonal stress due to serious problems with a family or friends or the death of a family member or close friend; Roberts et al., 2011). To support healthy relationships and prevent violence among couples the COVID-19 pandemic and onward, healthcare providers and mental health professionals should prioritize instilling healthy coping mechanisms to prepare people for stressful events (Bosó Pérez et al., 2021).

### Strengths and Limitations

There are several key considerations regarding interpretation of these data. First, while this was a large population-based survey using quota and weighting to achieve a quasi-representative sample, we are unable to make causal inferences with these cross-sectional data. As such, we cannot discern whether people’s fear of a partner led to deteriorations in their physical, mental, and social health or if the reverse is true. Some bidirectionality is likely; even though evidence we collected on people’s perceptions suggested specific negative outcomes of experiencing fear of a partner (Fig. 1), we did not ask whether those perceived correlates also led to IPV.

Second, direct comparisons between our prevalence estimates and those reported in other studies should be avoided or made with caution given methodological variations across studies. Because the remote collection of data about violence raised concerns regarding the risk of harm to participants and data quality, we decided to minimize the number and sensitivity of IPV items by only asking participants about their experiences of fearing a partner, which is also a strategy that is less confrontational than directly inquiring about specific IPV behaviors (Signorelli et al., 2022). For reasons regarding limited time, space, and trust, we measured fearing a partner as a proxy of IPV, whereas other studies may rely on more comprehensive behavior-specific assessments to examine specific types of IPV (Campbell et al., 2021; Costa et al., 2015). Despite these psychometric differences across studies and potential concerns that the threshold of fearing a partner might underestimate certain types of IPV (e.g., emotional, psychological, or financial types of aggression), our findings broadly corroborated prevalence estimates and sociodemographic correlates from a study of 30 countries that assessed specific types of IPV (Campbell et al., 2021).

Third, although self-reported measures provide insights to people’s IPV experiences (more so than domestic abuse hotline call data that do not provide context), people’s reports are subjected to several biases, especially for sensitive study topics. Of note, selection bias in the Natsal-COVID study may have resulted in an overrepresentation of people interested in sexual, reproductive, or relational health topics (Dema et al., 2022). Because this survey required participants to report experiences over the previous year, recall bias also limits the validity of these data. A further consideration is that people may have been even more likely to provide socially desirable responses if they were in a controlling relationship and may not have felt safe to report this or even take part in the survey; this may have particularly been the case where pandemic-related restrictions limited opportunities for people to be in a different space than their partner (Bosó Pérez et al., 2021). Finally, known sources of bias that may affect survey estimates exist in web-panel surveys compared with probability sampling methods (Dema et al., 2022); as such, inference of prevalence estimates in the general population should be done with caution.

### Implications and Future Directions

Our population-level estimates of fearing a partner and its health-related correlates during the COVID-19 pandemic in Britain emphasize harmful experiences that occurred alongside—and may have been exacerbated by—other wide-ranging hardships (e.g., disruption of health services during the pandemic). Despite the ubiquity of IPV and its negative health consequences for victims across numerous life domains, primary prevention of IPV has not been widely adopted in Britain (Sheng, 2020). In their review, Sheng noted that contemporary initiatives in the UK are education-based and designed to address gender inequality as well as promote respectful interpersonal relationships; yet, existing programmes remain small in scale and lack evidence regarding their effectiveness. Our findings suggest that efforts to promote gender inequality should consider specific groups of women who are most vulnerable to experiencing IPV (e.g., those with children at home or of lower socioeconomic status) and be complemented by tailored support for young people and sexual minorities of all genders.

In addition to efforts aimed at preventing IPV behaviors, there are opportunities for screening and detection (i.e., secondary prevention) as well as managing outcomes and rehabilitating (i.e., tertiary prevention). For example, secondary prevention efforts might include widespread usage of effective screening tools across healthcare settings—like the one on fearing a partner used in this study. IPV screening strategies should be particularly encouraged in sexual and reproductive health (SRH) settings because we found that fearing a partner was associated with greater odds of accessing SRH services. Of note, 51% of women who feared a partner and 44% of men who feared a partner accessed SRH services in the past year compared with 28% of other women and 9% of other men, respectively (see Appendix Table 3). Framed differently, 15% of women and 32% of men who accessed SRH services during this period reported fearing a partner. And because people are even more likely to disclose their IPV experiences within informal networks (e.g., friends, family) than they are to formally disclose to physicians or law enforcement professionals (Campbell et al., 2021), IPV detection and recovery efforts may also be improved by promoting healthy support networks and peer-based strategies (Paphitis et al., 2022).

Given our evidence that the health and wellbeing of those fearing their partner during the pandemic was much more likely to be worse than those who did not fear a partner, tertiary prevention efforts in the wake of the COVID-19 pandemic should address maladaptive coping mechanisms to allay further impairment (Bosó Pérez et al., 2021; Øverup et al., 2015). Services intended to manage the consequences of IPV and rehabilitate those affected may further benefit from telemedicine technologies that have generally advanced in the wake of the COVID-19 pandemic (Campbell et al., 2021). In these ways and others, there are multiple layers of approaches to assist people affected by IPV—with the ultimate goal being primary prevention.

### Conclusion

Our data suggest that a vulnerable group during the COVID-19 lockdowns comprised those fearing a partner—potentially due to intimate partner violence (IPV). These folks were especially at risk of being negatively affected by the pandemic; they also reported worse health and wellbeing outcomes a year after the first lockdown. Pathways from pandemic-related restrictions to increased occurrence or intensity of IPV might have included added stress inherent to economic insecurity, social isolation or perceived loss of control due quarantines, restricted availability of health services and access to first responders, or inability to temporarily escape abusive partners (Peterman et al., 2020). To care for those in relationships in which they fear their partner and avert additional IPV during pandemics, Peterman et al. (2020) recommended integrating IPV into healthcare responses, strengthening social safety nets, expanding shelter options for survivors, and encouraging informal (even virtual) social support networks.

Finally, this survey established a baseline investigation of the experiences associated with fearing a partner during the first year of the COVID-19 pandemic. As such, these findings will provide a useful reference point for subsequent studies (like the upcoming Natsal webpanel survey planned to take place in 2024). Not only will those comparisons provide a more complete understanding of IPV-related outcomes during lockdown periods, but they will also be critical for identifying groups with ongoing need following particularly negative experiences during the COVID-19 pandemic.

## Data Availability

The data are available from the UK Data Archive: 10.5255/UKDA-SN-8865-2.

## Appendix

Table 3

**Table 3:** Weighted percentages of accessing sexual or reproductive health services and associations with fearing a partner in the past year, by gender

	No partner fear in past year	Feared partner in past year	aOR^a^	*p*	aOR with rel. status^b^
Women					
Contraceptive services/advice	15.3 (14.1–16.7)	25.5 (20.6–31.1)	1.42 (1.05–1.94)	0.025	1.43 (1.04–1.98)
Fertility services/advice	1.2 (0.8–1.6)	8.1 (5.5–12.0)	5.92 (3.37–10.39)	< 0.001	5.71 (3.26-10.00)
Maternity/antenatal services	3.4 (2.8–4.2)	8.3 (5.5–12.5)	2.00 (1.19–3.33)	0.008	2.02 (1.20–3.42)
Abortion/pregnancy termination services	0.7 (0.5–1.1)	3.9 (2.2–6.7)	4.23 (2.07–8.65)	< 0.001	4.35 (2.13–8.89)
Cervical screening (smear test/pap test)	9.4 (8.3–10.5)	11.0 (7.8–15.2)	1.09 (0.73–1.62)	0.682	1.11 (0.74–1.67)
STI (sexually transmitted infection) testing	3.4 (2.7–4.1)	9.6 (6.5–13.8)	2.15 (1.34–3.46)	0.002	2.14 (1.27–3.62)
STI follow-up care	0.5 (0.3–0.8)	2.1 (0.9-5.0)	2.95 (1.08–8.11)	0.036	3.20 (1.11–9.19)
HIV testing	1.7 (1.2–2.3)	5.1 (3.0-8.7)	2.34 (1.21–4.53)	0.012	1.06 (0.96–4.03)
Advice or counselling for sexual problems	0.5 (0.3–0.8)	4.1 (2.2–7.2)	6.73 (2.79–16.19)	< 0.001	6.93 (2.77–17.34)
Relationship support services/advice	0.7 (0.4–1.1)	4.4 (2.5–7.9)	5.31 (2.25–12.53)	< 0.001	5.00 (2.10-11.92)
Sexual assault/rape support services or helplines	0.4 (0.2–0.7)	1.9 (0.8–4.2)	3.72 (1.28–10.84)	0.022	3.71 (1.28–10.83)
None	72.0 (70.3–73.7)	49.0 (43.0–55.0)	0.47 (0.36–0.62)	< 0.001	0.48 (0.37–0.63)
Denominator (unweighted, weighted)	2971, 2794	308, 276	—	—	—
Men					
Contraceptive services/advice	3.2 (2.5–4.1)	15.7 (11.4–21.3)	3.84 (2.36–6.27)	< 0.001	3.78 (2.26–6.32)
Fertility services/advice	1.5 (1.1–2.1)	8.3 (5.5–12.3)	3.89 (2.10–7.20)	< 0.001	3.08 (1.61–5.89)
STI (sexually transmitted infection) testing	2.9 (2.3–3.7)	11.8 (7.7–17.5)	3.02 (1.73–5.25)	< 0.001	2.95 (1.66–5.25)
STI follow-up care	0.9 (0.6–1.4)	7.8 (4.6–13.0)	6.27 (2.93–13.4)	< 0.001	5.30 (2.47–11.35)
HIV testing	1.6 (1.2–2.2)	8.0 (5.0-12.4)	3.61 (1.88–6.91)	< 0.001	3.68 (1.91–7.09)
Advice or counselling for sexual problems	0.8 (0.5–1.2)	6.7 (4.2–10.6)	6.45 (3.15–13.22)	< 0.001	5.99 (2.84–12.62)
Relationship support services/advice	0.8 (0.5–1.3)	7.7 (4.8–12.2)	7.13 (3.37–15.24)	< 0.001	6.14 (2.86–13.14)
Sexual assault/rape support services or helplines	0.3 (0.1–0.6)	6.5 (3.7–10.9)	14.64 (5.53–38.79)	< 0.001	12.06 (4.66–31.26)
None	91.1 (89.8–92.2)	56.4 (49.3–63.3)	0.18 (0.13–0.25)	< 0.001	0.20 (0.14–0.29)
Denominator (unweighted, weighted)	2634, 2792	241, 266	—	—	—

^a^Age-adjusted odds ratio. ^b^Age-adjusted odds ratio controlling for relationship status

95% confidence intervals are provided in parentheses

## Footnotes

1 ‘Women’ and ‘men’ each include trans women (unweighted *n* = 19) and trans men (unweighted *n* = 24), respectively, but not those who identify in another way.

2 We took the following actions mitigate potential risk to participants: provided an explicit “prefer not to say” option for each question, displayed reminders about the voluntary and confidential nature of the survey immediately before receiving the IPV items, and signposted relevant support services after the IPV questions and at the end of the survey.
